# **E**lectronic patient self-**R**eporting of **A**dverse-events: **P**atient **I**nformation and a**D**vice (eRAPID)**:** a randomised controlled trial in systemic cancer treatment

**DOI:** 10.1186/s12885-017-3303-8

**Published:** 2017-05-08

**Authors:** Kate Absolom, Patricia Holch, Lorraine Warrington, Faye Samy, Claire Hulme, Jenny Hewison, Carolyn Morris, Leon Bamforth, Mark Conner, Julia Brown, Galina Velikova, on behalf of the eRAPID systemic treatment work group

**Affiliations:** 1 Section of Patient Centred Outcomes Research (PCOR), Leeds Institute of Cancer and Pathology, University of Leeds, Leeds, UK; 2 Psychology Group, School of Social Sciences, Faculty of Health and Social Sciences, Leeds Beckett University, Leeds, UK; 3 Leeds Institute of Clinical Trials Research, University of Leeds, Leeds, UK; 4 Academic Unit of Health Economics, Leeds Institute of Health Sciences, University of Leeds, Leeds, UK; 5 Centre for Health Services Research, Leeds Institute of Health Sciences, University of Leeds, Leeds, UK; 6 Patient Representative, eRAPID systemic treatment workgroup, Leeds, UK; 7 Leeds Teaching Hospitals NHS Trust, St James’s Institute of Oncology, Leeds, UK; 8 School of Psychology, University of Leeds, Leeds, UK

**Keywords:** Cancer, Adverse events, Patient reported outcome measures (PROMs), Patient reported outcomes (PROs), Electronic patient records, Electronic health records, Internet, Intervention, Self-management, Chemotherapy

## Abstract

**Background:**

eRAPID (electronic patient self-Reporting of Adverse-events: Patient Information and aDvice) is an internet based system for patients to self-report symptoms and side effects (adverse events or AE) of cancer treatments. eRAPID allows AE reporting from home and patient reported data is accessible via Electronic Patient Records (EPR) for use in routine care. The system can generate alerts to clinical teams for severe AE and provides patient advice on managing mild AEs. The overall aims of eRAPID are to improve the safe delivery of cancer treatments, enhance patient care and standardise AE documentation.

**Methods:**

The trial is a prospective randomised two-arm parallel group design study with repeated measures and mixed methods. Participants (adult patients with breast cancer on neo-adjuvant or adjuvant chemotherapy, colorectal and gynaecological cancer receiving chemotherapy) are randomised to receive the eRAPID intervention or usual care over 18 weeks of treatment. Participants in the intervention arm receive training in using the eRAPID system to provide routine weekly adverse event reports from home. Hospital staff can access eRAPID reports via the EPR and use the information during consultations or phone calls with patients.

Prior to commencing the full trial an internal pilot phase was conducted (*N* = 87 participants) to assess recruitment procedures, consent and attrition rates, the integrity of the intervention information technology and establish procedures for collecting outcome data. The overall target sample for the trial is *N* = 504.

The primary outcome of the trial is quality of life (FACT-G) with secondary outcomes including health economics (costs to patients and the NHS), process of care (e.g. contacts with the hospital, number of admissions, clinic appointments and changes to treatment/medications) and patient self-efficacy. Outcome data is collected at baseline, 6, 12, 18 weeks and 12 months. The intervention is also being evaluated via end of study interviews with patient participants and clinical staff.

**Discussion:**

The pilot phase was completed in February 2016 and recruitment and attrition rates met criteria for continuing to the full trial. Recruitment recommenced in May 2016 and is planned to continue until December 2017. Overall findings will determine the value of the eRAPID intervention for supporting the care of patients receiving systemic cancer treatment.

**Trial registration:**

Current Controlled Trials ISRCTN88520246. Registered 11 September 2014.

## Background

Systemic drug treatments for cancer (chemotherapy, hormonotherapy, biological therapy, targeted agents) are associated with significant adverse events (AEs). An AE is an untoward symptom or disease associated with (but not necessarily causally related to) a medical treatment or intervention AEs may lead to changes in drug dosage, cessation of treatment and can significantly compromise patients’ quality of life. Severe AEs can escalate to hospitalisation for potentially life-threatening toxicities: 18% of cancer patients present to emergency services within 14 days of a scheduled hospital visit for symptom management (infection, fever, nausea/vomiting, pain, breathlessness) [1–4]. Patients with breast, gastrointestinal, colorectal cancers and those with metastatic disease are amongst those most likely to have emergency admissions [4, 5].

Many patients however, delay seeking care especially out of hours [3, 5]. This concurs with the findings of a UK enquiry into patient outcome and death (National Confidential Enquiry into Patient Outcome and Death, NCEPOD) which found that of patients dying within 30 days of systemic cancer therapy, 17% delayed seeking advice for over 24 h [6]. AEs are documented consistently by physicians in clinical trials however in routine care recording of AEs by clinicians and reporting by patients is variable and often omitted [6]. It has been recognised for some time that a structured AEs reporting system would be useful to facilitate correct documentation and grading of AE severity to support tailored management. Consequently, the National Cancer Institute (NCI) in the US have developed the Common Terminology Criteria for Adverse Events (CTCAE v 4.0) [7] as a reporting and severity grading system for cancer clinical trials. These have recently been adapted for patients to self-report (NCI-PRO CTCAE) [8] and these items have concordance with nurse evaluated AE [9] and similar items created for self-report correlate with quality of life measures [10]. The need for routine monitoring of cancer treatment AE is at odds with a health care system relying increasingly on patient self-management and home based care. In order to bridge the gap in service provision to detect, identify and manage AE in cancer patients the Electronic patient self-Reporting of Adverse-events: Patient Information and aDvice (eRAPID): system was developed [11].

### Patient reported outcome measures (PROMs)

PROMs have been used in clinical practice to support care of individual patients, recent reviews suggest they improve symptom/function monitoring, physician patient communication and decision making [12–17], can save time during clinic visits and improve the accuracy of symptom reporting [18]. In the UK the 2008 Darzi report [19] recommended that collection of PROMs data should be an essential component of health care evaluation [19] and the Department of Health (DOH) subsequently produced guidelines to aid their implementation [20]. Following this, use of PROMs in the health service is most advanced in England (particularly for performance comparisons) [21]. Two recently published reports by the Independent Cancer Taskforce and NHS England have continued to highlight the need to put PROMs at the centre of strategies to improve patient centred cancer care and quality of life [22, 23].

### Electronic and mobile reporting technology

Electronic reporting of patient reported outcome measures (PROMs) has proven extremely acceptable to patients in the clinic setting [24–26]. Examples of successful implementation of electronic symptom reporting in oncology clinical practice include PatientViewpoint [27], the symptom tracking and reporting system (STAR) system for patients to report chemotherapy AE [28] and the Tell Us™ [29] system for advanced cancer patients in hospices undergoing palliative care (all in the U.S.). In Austria the Computer-based Health Evaluation System (CHES) software [30] has been developed and an interactive online system (ISAAC) is in use in Canada [31]. In the UK the ASyMS mobile phone system is currently being evaluated [32]. Electronic patient reported outcome systems have proven very acceptable even for patients coping with extreme symptom burden and reduced quality of life; indeed a mean monthly PROM completion rate of 83% at 34 weeks has been achieved with patients receiving cancer treatment [33].

### eRAPID development work

**Fig. 1:**
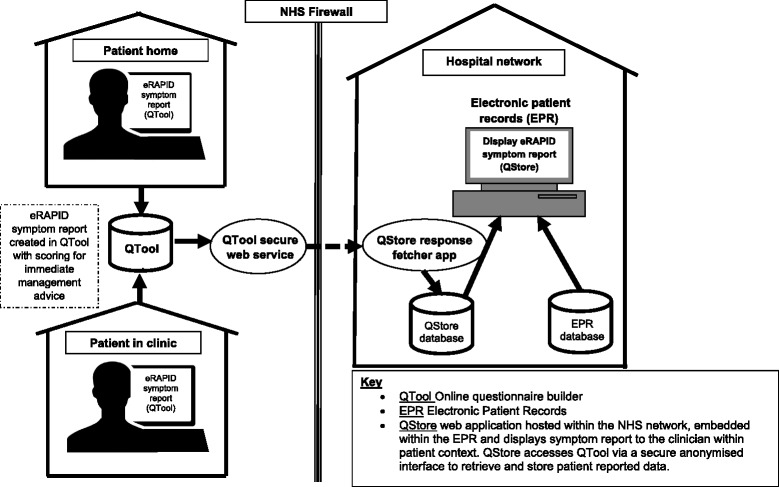
eRAPID system overview

The eRAPID research programme was designed to develop and evaluate an online system to support the collection and clinical integration of patients’ symptom/AE reports during cancer treatment. It utilises a web-based questionnaire builder system called QTool. QTool Version 1 was originally used in a large prospective study of cancer survivors, recruiting 636 patients in 12 months, 81% of whom completed web-based questionnaires at baseline [34] (www.epocs.leeds.ac.uk), confirming the feasibility of web-based patient-reporting and QTool acceptability. Between 2010 and 2013 the eRAPID developmental work was conducted (funded by an National Institute of Health Research grant: Programme Development Grant scheme RP-DG-1209-10,031), which focused on:

1. Developing the electronic platform to allow QTool data to be securely linked to the electronic patient records used by Leeds Teaching Hospitals (see Fig. 1).
2. Selection, adaption and evaluation of items for patients to report symptoms and AE resulting in the development of patient-reported AE (PRAE) items based on CTCAE grades [35]. The initial item pool includes most common AEs namely nausea, vomiting, diarrhoea, mucositis, fatigue, insomnia, palmar-plantar erythema, pain, peripheral neuropathy, appetite loss, constipation, rash, bleeding, anaemia, febrile neutropenia and stoma problems.
3. Collating patient information and advice on AE management***.*** We reviewed and compiled the extensive literature available providing patient advice on the management of common symptoms and side effects during systemic cancer treatment. The information is available on the password protected eRAPID patient website. The eRAPID QTool symptom report provides patients with immediate brief graded advice dependent on severity of AE reported (including a recommendation to contact the hospital when severe symptoms are detected) and links users out to the eRAPID website for more detailed information. The website has been extensively reviewed by both patients and oncology staff.
4. Mapping patient care pathways. With support from staff responsible for monitoring chemotherapy patients at St James’ Institute of Oncology, Leeds the current care pathways for patients receiving systemic treatment were mapped to establish where eRAPID can best fit. This work was conducted via: staff interviews, a local audit of care pathways/acute triage processes, mapping the existing chemotherapy pathways for the detection and management of AE and an assessment of patient experience of acute admissions and prospective patient interviews and diaries during chemotherapy to record AEs and costs to patents and services. The latter aimed to develop a questionnaire for health economic analysis [5].

This developmental work led to the:

- Successful mapping of current systemic treatment pathway, establishing where eRAPID is best placed
- Identification of staff requiring training to deliver eRAPID
- Adaptation of a health economic questionnaire for cancer patients receiving treatment

### The eRAPID intervention

**Fig. 2:**
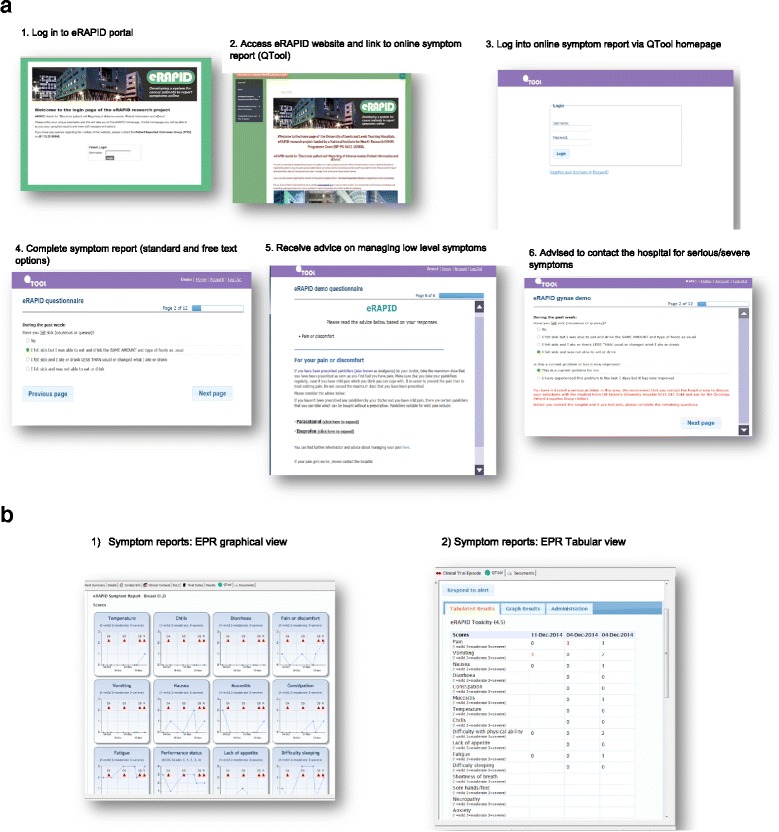
**a** Screenshots of eRAPID intervention (Patient login and symptom reports). **b** Screenshots of eRAPID intervention- Clinician view of symptom reports in electronic patient record (EPR)

An overview of the eRAPID intervention is described in Figs. 1, 2a and b. Figure 1 represents the technical components and their integration to support reporting of AEs immediately available in the EPR. The architecture protects patient confidentiality providing security whilst allowing immediate linkage to individual patient records to support care.

The intervention consists of the following components:

- Patients can log in to QTool (using a unique username and password) to access the eRAPID symptom questionnaire anywhere with internet access (including home or hospital).
- For mild/moderate problems information about self-managing these issues are provided via brief instructions in QTool along with hyperlinks to more detailed advice on the eRAPID patient website (Fig. 2a).
- Where severe symptoms are reported patients are advised to contact the hospital.
- The patient reported data is immediately available for staff to view in the individuals’ electronic patient records in Leeds Teaching Hospitals NHS Trust (Patient Pathway Manager, PPM). See Fig. 2b.
- Alerts for severe symptom reports are sent directly to staff via email. Clinicians can then log into PPM and view the patients’ symptom reports and take appropriate action where needed.

Prior to the start of the current trial the eRAPID system underwent usability testing with *N* = 14 breast cancer patients receiving adjuvant or neo-adjuvant chemotherapy and relevant staff.

### Hypotheses

We hypothesise the eRAPID intervention has the potential to bring benefit to patients, staff and the NHS in the following ways:

- Benefits for patients
  - ○ Earlier symptom detection and improved self-management, timely admissions
  - ○ Improved supportive medication use
  - ○ Appropriate hospital, GP, community contacts
  - ○ Better outcomes (improved symptom control, functioning and quality of life)
- Benefits for staff
  - ○ Reduce the number of hospital, GP, community contacts
  - ○ Save time spent on enquiring and recording AEs
  - ○ Focus attention during clinical contacts on most important or severe AEs
  - ○ Support decision making in routine care
- Benefits to the NHS
  - ○ eRAPID provides a cost-effective approach to support patient self-management and reduce hospital and GP contacts

### Study design

This study is a single centre 1:1 allocation prospective randomised two-arm parallel group trial design with repeated measures and mixed methods.

### Patient sample

The study sample includes patients with gynaecological or colorectal cancer requiring chemotherapy, or breast cancer undertaking either neo-adjuvant or adjuvant following systemic treatment pathways at St. James’s Institute of Oncology, Leeds, UK.

## Methods

**Fig. 3:**
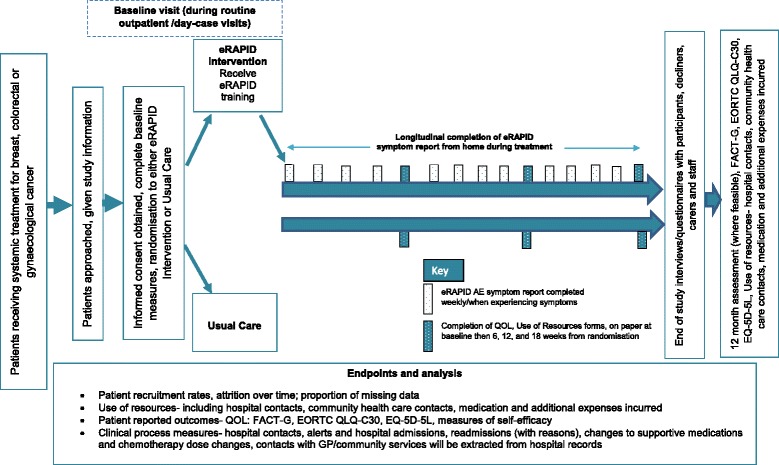
Trial flow diagram

Participants are randomised to either the intervention arm (eRAPID plus usual care) or the control arm (usual care). See Fig. 3 for the trial flow diagram. Participants are on the study for an 18 week period from the start of chemotherapy. A subset of participants (where feasible within the funding timeframe) will also be assessed at a 12 month time point to examine any potential longer term impact of the intervention on quality of life and clinical processes.

### Usual care

Includes an initial consultation with an oncologist to decide whether to commence systemic treatment. Patients are provided with verbal and written information on treatment benefits and expected AEs, and are given instructions on how to contact the hospital. They have a nurse assessment before starting their treatment. During treatment patients are routinely assessed in clinics for AE and to prescribe their next cycle of treatment by an oncologist, Clinical Nurse Specialist (CNS) or staff grade doctor. Depending on AE experienced by the patient, treatment doses can be reduced, and/or supportive medications changed (e.g. anti-sickness drugs, anti-diarrhoea drugs). When at home if patient has a serious AE they are asked to contact the hospital and the nurse dealing with the patient phone call uses an Acute Triage Form to record reasons for the call, document the AE and gives advice.

### eRAPID intervention

In addition to usual care, participants randomised to the eRAPID intervention arm will receive training on using the system and will be asked to complete the eRAPID symptom report routinely from home at least weekly and when they experience symptoms over 18 weeks during treatment. Clinicians are given access to patients’ self-reported AEs via the electronic patient record system (PPM) and asked to utilise the information when seeing patients in clinic or providing telephone advice.

### Aims and study objectives

To evaluate the potential benefits of eRAPID for patients and staff, the intervention and usual care arm will be compared on the following areas through the collection of appropriate clinical information, patient reported outcomes and interview data:

1. Assessment of hypothesised benefits to patients with mild or moderate AE:

- Number of hospital, GP and community contacts during the study
- Improved patient reported outcomes
- Improved symptom detection and supportive medication use

2. Assessment of hypothesised benefits to patients with severe AE:

- Improved detection and treatment of AEs and admissions (e.g. number of clinician alerts generated from eRAPID, number of admissions and hospital contacts)
- Levels of morbidity (percentage of planned chemotherapy received, changes to treatment plans (dose reductions, dose delays/interruptions)).

3. Assessment of hypothesised benefits to clinicians: Staff will be interviewed about their views of the value of eRAPID in saving time currently spent enquiring and recording patients’ AE and supporting treatment decision-making. In addition oncologists will complete a feedback form at routine review appointments after seeing eRAPID intervention participants to assess how/if patient reports are used.
4. Monitor patient safety, assessed by monitoring acute admissions, cumulative deaths and cause of death.

The FACT-G Physical Wellbeing Score [36] (measured at 18 weeks) is the primary outcome. The main secondary outcome is cost effectiveness assessed via use of health care services (including hospital admissions, telephone contacts and consultations, medication and personal expenses). In addition participant records will be linked to costs held within the local pilot database of the National Patient-Level Information and Costing System (PLICS) scheme. This provides a cost for hospital based accident and emergency department visits, outpatient attendances and inpatient stays.

### Ethical approval

The study was approved by the National Research Ethics Service (now part of the Health Research Authority) Yorkshire & The Humber Leeds East Committee in September 2014 (Reference 14/YH/1066). Local approvals from the Leeds Teaching Hospitals NHS Trust Research and Innovation Department were also obtained.

### The RCT has two phases

I. An internal pilot phase to assess the feasibility and acceptability of the intervention and allow for minor modifications before further large scale recruitment was conducted. If no meaningful changes are made to the intervention the study would progress to the main trial and patients recruited during the pilot phase will be included in the analysis.
II. The full trial phase will continue to recruit the target sample (at most *N* = 504 participants, see sample size calculation below) using the best recruitment and retention methods established in the internal pilot.

### Internal pilot phase

Prior to starting the full trial an internal pilot phase was conducted with the aim of assessing recruitment and attrition rates, refining the intervention, testing the integrity of information technology (IT) systems and to establish procedures and methods for collecting outcome measure data. We aimed to achieve (i) recruitment levels of >10 patients per month), (ii) 60% to consent to randomisation, and (iii) <30% attrition.

The pilot sample size was set at 30 participants per-arm [37] allowing for 30% overall attrition, the overall target was a minimum of 42 patients per-arm (*N* = 84). Recruitment took place between January–September 2015.134 patients were approached, 87 consented, 22 declined and 25 were excluded after further screening (no Internet access or not continuing on to chemotherapy). The consent rate when including those patients excluded post-screening was 65% (87 consented/134 approached). However the “true” consent rate excluding the 25 patients was 80% (134 approached - 25 ineligible). Forty-four participants were allocated to the Intervention arm and 43 to Usual Care. Only 13 participants (15%) withdrew. No significant problems with the IT systems underpinning the eRAPID online intervention were encountered and the research team was able to develop robust methods of gathering information on clinical process data (e.g. hospital contacts, changes to treatment). Based on participant feedback some refinements were made to patient “use of resources forms” to aid comprehension of questions and ease of completion. The overall recruitment and attrition targets were met and the Trial Steering Committee (TSC) recommended progression to the main trial. The study procedures described below reflect the protocol for the main trial approved by Yorkshire & The Humber Leeds East Research Ethics Committee in December 2016, protocol version number 1.5.

### Patient eligibility

#### Inclusion criteria

- Adult patients (aged 18 years or over) attending St James’ Institute of Oncology, Leeds with breast cancer undertaking either neo-adjuvant or adjuvant systemic treatment pathways, gynaecological or colorectal cancer requiring chemotherapy
- Prescribed at least 3 months of planned chemotherapy cycles at the time of study consent
- Able and willing to give informed consent
- Able to read and understand English
- Access to the internet at home

#### Exclusion criteria

Patients are excluded from participation if they are:

- Taking part in other clinical trials involving the completion of extensive patient reported outcome or quality of life measures or have previously participated in an eRAPID trial
- Exhibiting overt psychopathology/cognitive dysfunction

### Recruitment processes

#### Identification of eligible patients

Patients are recruited from outpatient clinics and day case wards at St James’ Institute of Oncology clinics.

Eligible patients are identified by screening of the clinic, in-patient or day-case lists by the most appropriate clinical staff. Prior to study commencement, consultants responsible for the care of patients within each eligible tumour group are contacted via email and sent an introduction to the study and permission is requested for the research team to approach their patients.

#### Approaching patients

An appropriate member of the clinical team seeks permission from eligible patients for the researcher to speak to them about the study. After introduction from clinical staff, eligible patients are approached by a member of the research team who explain the study and provide the information sheet. Patients are given as much time as they need to read the information and ask questions and should they wish to participate they are consented at the visit. Where patients prefer more time to consider participation, they can take the information home and discuss the study again with the researcher at their next visit.

When patients are happy to participate they are asked to provide written informed consent. The participant is then randomised to either the intervention or control arm. Participants who are randomised to the intervention arm receive training in using the eRAPID system.

### Randomisation

**Fig. 4:**
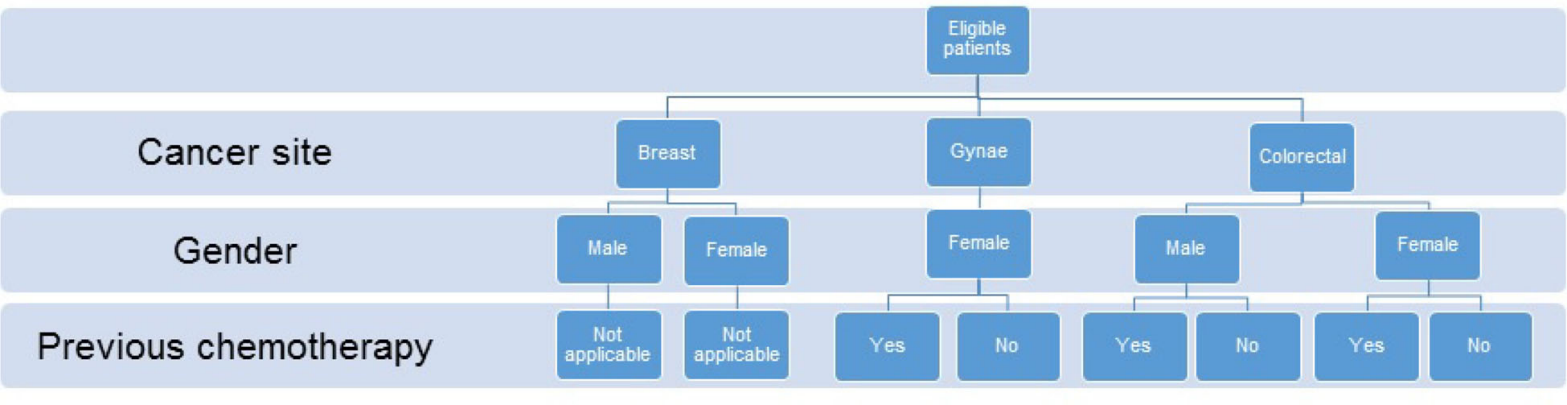
Stratification factors used in randomising patients in the eRAPID RCT

After trial eligibility has been confirmed and consent given, randomisation is performed via the University of Leeds Clinical Trials Research Unit (CTRU) telephone system. Participants are randomised with 1:1 allocation to intervention and control groups. Patients are stratified by cancer site (breast, gynaecological or colorectal), gender and previous chemotherapy (gynaecological cancer patients only) in variable random permuted blocks of 4, 6 or 8, see Fig. 4.

### eRAPID intervention: Participant and staff training

#### Participant training

Researchers provide a short demonstration on how to use the eRAPID system and provide patients with a unique user name and password to access the system, on an eRAPID ‘postcard’. Participants are given a user manual to take home providing a step-by-step guide on how to log in and use the eRAPID system. Participants are asked to complete the remote eRAPID Adverse Events (AEs) questionnaire weekly (from home or during clinic visits) and at any time when they experience any side-effects/symptoms during the duration of their treatment. The questionnaire consists of 12–15 items depending on the disease group assessing the severity of common symptoms such as: nausea, vomiting, pain, fatigue, diarrhoea, constipation, sore mouth/tongue, temperature, chills, performance status, fatigue, sleep, and appetite. Participants can also provide details about additional problems at the end of the standard questions. A weekly text message or email reminder are sent to the participants as a prompt to complete the eRAPID AE questionnaire.

#### Staff training

Prior to study commencement the appropriate staff received training on eRAPID. The aims of training are to support staff in understanding:

1. How patients use and interact with eRAPID and the content of self-reported AE questionnaire/website
2. Accessing patients’ eRAPID self-report data in the electronic patient records
3. Interpreting patient self-reported AE scores and methods of incorporating the data into clinical encounters with patients. Including information on how the symptom scores relate to mild, moderate and severe problems and how the cut-offs or alerts for severe symptoms have been developed

During one-to-one/small group interactive sessions eRAPID is demonstrated by the research team, giving staff an opportunity to see the patient interface. Staff are shown the practicalities of locating the data within the electronic patient records. Manuals are provided outlining the key steps in all the processes covered in the session. Training highlights that the self-report information should be seen as a supplementary resource for staff to use in conjunction with routine practices for clinical decisions.

### Outcome measures

The following measures and data are being collected to enable comparison between the usual care and eRAPID intervention arms. An overview of the outcome data and time points are outlined in Tables 1 and 2.

**Table 1:** eRAPID RCT in systemic cancer treatment: Participant completed primary and secondary outcomes measures

Questionnaire title and brief description	Item information/response format and scoring	Example questions	Time points
Primary outcome- Quality of Life			
Quality of life: FACT-G [36]			
27 item cancer specific QOL measure four subscales covering physical, social or family, emotional and functional wellbeing	5 point scale (0 not at all – 4 very much)	• I have nausea	Baseline, 6, 12, 18 weeks and 12 months
		• I am forced to spend time in spend	
	Higher subscale and total scores indicate better QOL (score range 0–108).	• I get support from my friends	
		• I worry that my condition will get worse	
		• I have accepted my illness	
Secondary outcomes- health economic/clinical process data			
EQ-5D-5 L [38]			
6 item descriptive health profile (measuring mobility, self-care, usual activities, pain, anxiety/depression) and a single index value for health status that can be used as part of a health-economic evaluation.	5 items measured on 5 point scale and single global health item rated from 0 (worst health) to 100 (best health)	Self-care	Baseline, 6, 12, 18 weeks and 12 months
		• I have no problems washing of dressing myself	
		• I have slight problems washing or dressing myself	
		• I have moderate problems washing or dressing myself	
		• I have severe problems washing or dressing myself	
		• I am unable to wash or dress myself	
Use of Resources			
Assessment of financial impact of cancer treatment covering:	Varied tick boxes and free text options.	• Please complete the boxes below to tell is about any non-hospital health care contacts you have had in the last 6 weeks	6, 12, 18 weeks and 12 months
- Employment status			
- Contacts with community health care services (GP, district nurses etc)			
- Medications costs		• Please tell us about any medications you have been prescribed in the last 6 weeks and who prescribed it	
- Cancer related travel costs			
- Cancer related food/drink costs			
- Additional expenses			
		• Please tell us about any additional travel costs related to your cancer or cancer treatment you have incurred in the last 6 weeks	
EORTC-QLQ C30 [39]			
30-item questionnaire with five functional scales (physical, emotional, cognitive, social, role), three symptom scales (fatigue, pain, nausea/vomiting), a global health related quality of life scale, and six single items (anorexia, insomnia, dyspnoea, diarrhoea, constipation, financial difficulties)	Questions are rated on a 4 or 7 point response scales.	• Do you have any trouble taking a long walk	Baseline, 6, 12, 18 weeks and 12 months
		• During the past week…	
	The scales and single-item responses are recalculated into a score from 0 to 100.		
		- Have you lacked appetite?	
	• A high functional scale score represents a high level of functioning	- Were you tired?	
		- Did you feel depressed?	
	• A high score for the global health status/QOL represents a high QOL		
	• A high score for a symptom scale/item represents a high/worse level of symptomatology		
Secondary outcomes- Self-efficacy			
Self-Efficacy for Managing Chronic Disease [34]			
6-Item scale covering several domains common across chronic diseases (symptom control, role function, emotional functioning and communicating with physicians)	Items rated from 1- (not at all confident) to 10 (totally confident)	• How confident are you that you can keep physical discomfort or pain of your disease from interfering with the things you want to do?	Baseline and 18 weeks
	The score for the scale is calculated from the mean of the six items.	• How confident are you that you can do things other than just taking medication to reduce how much you illness affects your everyday life?	
Cancer Behaviour Inventory-Brief (CBI-B) [40]			
A measure of self-efficacy for coping with cancer. 14 items (adapted from full 33 item measure)	Items are rated on a 9-point scale ranging from 1 (“*not all confident*”) to 9 (“*totally confident*”)	Please read each numbered item. Then rate that item on how confident you are that you can accomplish that behaviour.	Baseline and 18 weeks
		- Maintaining independence	
		- Expressing feelings about cancer	
	A total score is calculated as the sum of all 12 items.	- Asking physicians’ questions	
		- Coping with physical changes	
Patient Activation Measure (PAM) [41]			
13-item scale for measuring the level of patient engagement in their healthcare (knowledge, skill and confidence for self-management)	Statements rated on 4 point scale from disagree strongly to agree strongly and additional N/A option.	• When all is said and done I am the person who is responsible for taking care of my health	Baseline, 18 weeks and 12 months
		• I am confident I follow through on medical treatments I may need to do at home	
	Responses are combined to provide a single score of between 0 and 100 with higher scores representing higher levels of patient activation.	• I know what treatments are available for my health problems.	
	Scores can be classified into one of four groups, known as ‘levels of activation’.		
Secondary outcomes- eRAPID/IT system performance			
System Usability Scale (SUS) [42]			
10 item instrument to assess views of usability of an IT systems.	Each statement rated from 1 strongly disagree to 5 strongly agree.	• I think that I would like to use this system frequently	18 weeks
		• I thought there was too much inconsistency in this system	
	Responses are calculated into a total score ranging from 0 to 100 with higher scores representing better system usability.	• I felt very confident using the system	
eRAPID end of study questionnaire			
15 statements/free text boxes to assess participant views of using eRAPID and suggestions for improvements	Statements rated on 3–5 response option scales (e.g. very easy-very difficult) and free text boxes for comments.	• How easy or difficult was it to learn how to use the eRAPID system?	18 weeks
		• How did you feel about the amount of time it took to complete the symptom questions?	
		• To what extent do you feel that the symptom questionnaire was useful for the doctors and nurses you saw during your treatment?	
		• Have you got any suggestions about how the eRAPID system could be improved?

**Table 2:** eRAPID RCT in systemic cancer treatment: Researcher collected data for secondary outcomes

Data	Description of data	Time point for collection
Treatment and clinical information	• Cancer diagnosis, stage and grade	Initial baseline assessment and reviewed for changes at 18 weeks
	• Age, date of birth	
	• Baseline data on planned chemotherapy	
	• Changes to treatment delivery and reason	
	• Comorbidities	
Clinical process- Hospital contacts	• Contacts with the hospital e.g. (unplanned) telephone, appointments, consultations	• Data extracted from medical notes for 18 of study
	• Emergency admissions, acute ward stays and reasons for contacts.	
		• 3 month prior to 12 month follow-up assessment
Clinical process- Information from general practice	• GP recorded problems/concurrent illnesses	• Data extracted from medical notes for 18 week study period
	• Prescribed medications and reasons for prescription (where available)	
		• 3 month period prior to 12 month follow-up assessment for subset of participants
IT/System functioning	• Researcher maintained log of IT issues (e.g. server downtime, contacts with study participants reporting IT problems or issues logging into eRAPID) and how these were resolved	Throughout trial
Treatment and clinical information	• Cancer diagnosis, stage and grade	Initial baseline assessment and reviewed for changes at 18 weeks
	• Age, date of birth	
	• Baseline data on planned chemotherapy	
	• Changes to treatment delivery and reason	
	• Comorbidities	
Clinical process- Hospital contacts	• Contacts with the hospital e.g. (unplanned) telephone, appointments, consultations	• Data extracted from medical notes for 18 of study
	• Emergency admissions, acute ward stays and reasons for contacts.	• 3 month prior to 12 month follow-up assessment
Clinical process- Information from general practice	• GP recorded problems/concurrent illnesses	• Data extracted from medical notes for 18 week study period
	• Prescribed medications and reasons for prescription (where available)	
		• 3 month period prior to 12 month follow-up assessment for subset of participants
IT/System functioning	• Researcher maintained log of IT issues (e.g. server downtime, contacts with study participants reporting IT problems or issues logging into eRAPID) and how these were resolved	Throughout trial

### Patient outcome measures

#### Functional assessment in cancer therapy scale-General (FACT-G) [36]

The FACT-G is a cancer specific measure widely used in clinical trials. It has four subscales: physical wellbeing, social or family wellbeing, emotional wellbeing, and functional wellbeing. Question responses range from 0 to 4. Higher scores on the questionnaire indicate better quality of life.

#### Eq-5D-5 L [38]

The EQ-5D is a standardised instrument for use as a measure of health outcome developed by the EuroQol Group. The instrument assesses five dimensions: mobility; self-care; usual activities; pain/discomfort and anxiety/depression. Each dimension has five response levels (ranging from no problems to extreme problems). The instrument also includes a scale to rate health from 0 (worst health you can imagine) to 100 (best health you can imagine).

#### Use of resources

Resource use is assessed using patient forms (detailing non-hospital contacts e.g. appointments with GPs/community services, counsellors, local support services), as well as medication use and costs incurred as a consequence of cancer diagnosis/treatment. This form is based on those developed by Hulme for a recently completed trial assessing treatment for chemotherapy-related nausea/vomiting (https://njl-admin.nihr.ac.uk/document/download/2002381).

#### EORTC-QLQ-C30 [39]

The EORTC QLQ-C30 is a 30-item questionnaire consisting of five functional scales (physical, emotional, cognitive, social, role), three symptom scales (fatigue, pain, nausea/vomiting), a global health related quality of life scale, and six single items (anorexia, insomnia, dyspnoea, diarrhoea, constipation, financial difficulties). Questions are rated on a 4 or 7 point response scale and overall scale scores are calculated from 0 to 100 with higher scores indicating better quality of life or functioning. Symptoms scales are scored so that higher scores indicate worse symptoms experience.

### Self-efficacy and patient activation

#### *Self-Efficacy for Managing Chronic Disease 6-Item Scale* [34]

This 6-item scale covers several domains that are common across many chronic diseases such as symptom control, role function, emotional functioning and communicating with physicians.

#### *The Cancer Behaviour Inventory- Brief (CBI-B)* [40]

A self-efficacy measure specifically designed for assessing coping with cancer. Devised from the full 33 item measure, this brief version has 14 items covering: maintaining activity and independence, seeking and understanding medical information, stress management, coping with treatment related side effects and accepting cancer/maintaining a positive attitude.

#### *The Patient Activation Measure (PAM)* [41]

The PAM is a tool for measuring the level of patient engagement in their healthcare. It was designed to assess an individual’s knowledge, skill and confidence for self-management. The PAM 13-item scale explores beliefs, knowledge and confidence for engaging in health behaviours. Each item is rated on a four point scale from strongly disagree to strongly agree and an overall score from 0 to 100 can be calculated. These scores can be subdivided to categorise people into one of four activation categories ranging from 1- Low activation to 4- High activation.

### Socio-demographic and clinical process data

Participants complete a baseline questionnaire on socio-demographics and current computer usage. Clinical baseline data are obtained from participants’ medical notes and include diagnosis, co-morbidities and planned treatment (Table 2).

To determine any association between the eRAPID intervention and improved detection and management of AEs, data is collected from hospital triage forms, medical records, hospital databases to record:

- Number of scheduled and unscheduled hospital contacts (admissions, clinic visits, phone calls with staff)
- Changes to supportive medications and chemotherapy dose changes
- Contacts with GP and community services
- Number of clinician alerts generated from eRAPID severe symptom reports and actions taken by staff

### eRAPID system performance

Throughout the study the eRAPID IT system is monitored for unscheduled server down time (leading to the unavailability of the QTool questionnaire website, eRAPID website and patient symptom data in PPM). A log of phone calls/feedback from study participants regarding issues/problems surrounding the use of the eRAPID questionnaire or website will be maintained.

eRAPID intervention participants are asked to complete the System Usability Scale [42] (SUS). This 10 item instrument assesses subjective views of usability of different systems including hardware, software, mobile devices, websites and applications. The 10 items cover the ease of using the system, its complexity and user confidence. Each item is rated from 1 to 5 and a composite score of overall usability can be calculated ranging from 0 to 100 (higher scores reflect better usability). Intervention participants are also asked to complete a short end of study questionnaire about their experiences with the eRAPID intervention which includes free text boxes for comments and feedback.

### Participant interviews

Between 5 and 10 participants per disease group and study arm will be interviewed at the end of the full trial. Participants will be asked about their treatment experience, how they managed and monitored their symptoms and perceptions of reporting and discussing their symptoms with hospital staff. Intervention arm participants will be asked to describe their thoughts on using the eRAPID system.

### Staff feedback- interviews and questionnaires

At routine chemotherapy review appointments involving eRAPID intervention patients, staff will be asked to provide:

- Clinician reports of use of eRAPID patient data during consultations
- At 6 weeks routine clinic visits clinicians are asked to complete CTCAE scoring form matching those AE completed by patients on the eRAPID questionnaire

At the end of the study 5 health professionals from each disease group will be interviewed to determine their views of eRAPID, the perceived value and use of the patient data in clinical practice (e.g. improving the detection, documentation and management of AE, supporting treatment decision-making in routine care). Perceptions of staff training needs and recommendations for improving the system will also be explored.

### Sample size calculations

The sample size for the full trial is based on the primary patient outcome of better symptom control measured at 18 weeks by the FACT-G. A sample of 176 patients per arm is necessary to detect a 2-point change in the FACT-G Physical Wellbeing score with 80% power and 5% significance, where the population standard deviation is 6.7. This change corresponds to a medium Cohen’s effect size (0.3) [43].

Allowing for 30% attrition, a minimum of 252 patients per arm (504 total) is required. With potentially >500 eligible patients treated in the cancer centre annually, we expect to recruit 20 patients per month over approximately 24–30 months, allowing for 70% internet access and 70% consent rate.

### Analysis populations

All analyses and data summaries will be conducted on the intention-to-treat (ITT) population which is defined as all participants registered regardless of non-compliance with the protocol or withdrawal from the study.

### Statistical analysis

#### Baseline characteristics

Data from the baseline socio-demographic, computer usage and clinical data questionnaires will be tabulated using frequencies and summary statistics for each treatment group and overall for both the pilot phase and full trial.

### Primary outcome

The FACT-G Physical Well-being score will be summarised overall and by treatment arm. Changes in score over time and differences between treatment arms will be explored using a multilevel repeated measures model. The model for each post-randomisation point will be adjusted for baseline score and stratification factors. If there are missing items, subscale scores will be prorated as per the FACT-G scoring manual.

### Secondary outcomes

#### Clinical process measures

The number of calls made to the hospital will be summarised overall and by treatment arm. Differences between the two treatment groups will be compared using either Poisson regression or negative binomial regression; the most appropriate model will be chosen after performing post-estimation tests. Models will be adjusted for the stratification factors.

The numbers of weekly/additional AE reports and severe AE alerts generated will be summarised for participants randomised to the eRAPID intervention. The number of telephone calls to hospital staff, acute admissions, contacts with GP and/or community services and number of deaths will be summarised overall and by treatment arm. Any differences between treatment arms will be explored using the most appropriate regression model (either Poisson or negative binomial, to be decided using post-estimation tests) adjusted for stratification factors.

#### Patient outcome measures (other than primary)

Changes in scores over time and differences between treatment arms will be explored using a multilevel repeated measures model adjusted for baseline scores and stratification factors. As the sample size was not powered to detect changes in these outcome measures, statistical significance will be assessed at the 1% level.

### Health-economic data

An embedded health-economic study will allow within trial incremental cost-effectiveness analysis (18 weeks) taking the perspective of the service provider including the costs of NHS and Personal Social Services. The analysis will compare usual care with the eRAPID-supported pathway. A secondary analysis will take a societal perspective. Analyses will use quality-adjusted-life-years (QALYs) outcome-measures. Estimation of QALYs requires the production of utility-weights for each health-state observed in the trial population. We will use the EQ-5D-5 L for this purpose [3, 44] collected at baseline, 6, 12 & 18 weeks. We will also use EORTC QLQ-C30 to derive utilities (EORTC QLQ-U10) to calculate QALYs in the same way. This will limit the need to interpolate quality of life between observation points [45]. NHS resource-use associated with each treatment modality will be collected using the process-of-care measures to contribute to a health-economics analysis of additional health financial costs related to treatment and the study. Use of outpatient and community-based health and social care (including, for example, home help or residential care) will be collected from the patient at baseline, 6, 12, and 18 weeks with the Use of Resources questionnaire developed in the Programme Development Grant and tested in the pilot study. Unit financial costs for health services resources will be obtained from national source: the Personal Social Services Research Unit, the British National Formulary and NHS reference cost database [46–48]. Given the duration of the trial discounting is not required.

Secondary analysis will include costs to participants (travel expenses, over the counter medicines) and productivity losses.

In addition to the analyses at 18 weeks we will undertake an exploratory cost effectiveness analysis (including a planned a–priori sub-group cost-effectiveness analysis at 12 months using a sub-sample of participants for whom we have collected resource use, EQ-5D-5 L and EORTC QLQ-C30 data).

For each analysis we will undertake probabilistic sensitivity analysis using bootstrapping. The results will be presented as the Expected Incremental Cost Effectiveness Ratio, scatter plot on the cost-effectiveness plane and a Cost Effectiveness Acceptability Curve. We will calculate the expected net-benefit assuming lambda has a value of £20,000 [49].

### Qualitative data

Interviews will be recorded and transcribed. Data will be managed by NVivo software and analysed using thematic analysis [37, 50]. Two researchers will independently look for the emerging themes and code them. Then they will meet, compare the codes/themes and resolve any potential conflicts by consensus.

## Discussion

This paper describes the protocol for the eRAPID RCT in systemic cancer treatment. eRAPID is a unique web based intervention designed to improve the systematic reporting of AE during cancer treatment and improve patient care and experiences. A number of web based PROMs systems have been developed. Since the current trial began Basch and colleagues in the U.S. have published findings from an RCT using the STAR (Symptom Tracking and Reporting) web interface during chemotherapy indicating a positive impact on patients’ quality of life, treatment delivery, number of emergency room attendances and 1 year survival [44]. We believe eRAPID is the first of its kind to allow remote monitoring of symptoms and side effects where patient reported data is accessible alongside standard clinical information in electronic patient records as well as providing patients with immediate symptom management advice. We hypothesise that these features along with alerts for severe symptoms will lead to improved clinical outcomes for participants allocated to the eRAPID intervention and will benefit health care services.

This study can be considered a complex intervention due to the number of active components involved. These include the new technology for patients completing symptom self-reports from home, automatic advice on managing mild symptoms and when to contact the hospital for severe problems, the availability of this patient data for staff to use in clinical practice, alert generation for severe problems and maintaining staff training and engagement. Consequently eRAPID’s success relies on the investment of both staff and patient groups in the intervention and the robustness of the IT supporting the system. Although the eRAPID website and the online symptom reporting questionnaire have undergone extensive usability testing, the pilot phase of the RCT was considered vital in order to assess the intervention over a longer time frame and with all participating cancer groups as each differ in terms of the care pathways and staff involved. The decision to perform an internal pilot, rather than a separate pilot study, was motivated by our intention to avoid losing momentum and reduce the time between the end of the pilot and the start of the main trial [45]. This approach aimed to maintain continuity with the staff involved in the eRAPID intervention both in terms of recruitment and utilising the patient AE reports in clinical encounters.

The study is funded as part of 5 year programme, in parallel we are developing multi-centre eRAPID interventions for cancer patients receiving radiotherapy and surgery which will be evaluated in separate pilot studies. If found to have a positive effect on patient wellbeing and use of health care resources, eRAPID has the potential to provide a cost effective enhancement to the standard care of cancer patients. Such an approach could also be extended to long-term survivorship beyond cancer treatment [49].
